# Supplementary material for: Synthesis and Characterization of a Mg2+-Selective Probe Based on Benzoyl Hydrazine Derivative and Its Application in Cell Imaging
Source: Molecules. 2021 Apr 23;26(9):2457. doi: 10.3390/molecules26092457 (PMC8122791; doi:10.3390/molecules26092457)
Supplement: Supplementary file 1 [file molecules-26-02457-s001.zip › molecules-1136697-supplementary.pdf]

Synthesis and Characterization of a Mg^{2+}-Selective Probe Based on Benzoyl Hydrazine Derivative and Its Application in Cell Imaging

Chunwei Yu [1], Yuxiang Ji [1], Shaobai Wen [1] and Jun Zhang [1,2,*]

[1] Laboratory of Environmental Monitoring, School of Tropical and Laboratory Medicine, Hainan Medical University, Haikou 571101, China; cwyu1979@163.com (C.Y.); jiyuxiang@hainmc.edu.cn (Y.J.); wenshaobai@163.com (S.W.)
[2] Laboratory of Tropical Biomedicine and Biotechnology, Hainan Medical University, Haikou 571101, China
* Correspondence: jzhang@hainmc.edu.cn; Tel.: +86-898-66973160

Figure S1 ESI-MS mass spectrum of **P**.....2
Figure S2 IR spectrum of **P**.....3
Figure S3 ^{1}H-NMR spectrum of **P**.....4
Figure S4 ^{13}C-NMR spectrum of **P**.....5
Figure S5 (a) fluorescence response of **P** (10 μM) to 10 μM of Mg^{2+} and to the mixture of 10 μM individual other metal ions with 10 μM of Mg^{2+}; (b) fluorescence response of **P** (10 μM) to 10 μM of Mg^{2+} and to the mixture of 10 μM individual anions with 10 μM of Mg^{2+}.....6
Figure S6 The plot of fluorescence intensity at 482 nm shows a linear relationship of $1/(F - F_0)$ vs $1/[Mg^{2+}]$, indicating the 1:1 stoichiometry of Mg^{2+} and probe **P**.....7
Figure S7 ESI-MS mass spectrum of **P + Mg^{2+}**.....8
Figure S8 ^{1}H-NMR spectrum of **P + Mg^{2+}**.....9
Figure S9 IR spectrum of **P + Mg^{2+}**.....10
Figure S10 Cell viability values (%) estimated by MTT test versus incubation concentrations of **P**. Hl-7701 cells were cultured in the presence of 0–10 μM **P** at 37 °C.....11

Figure S1

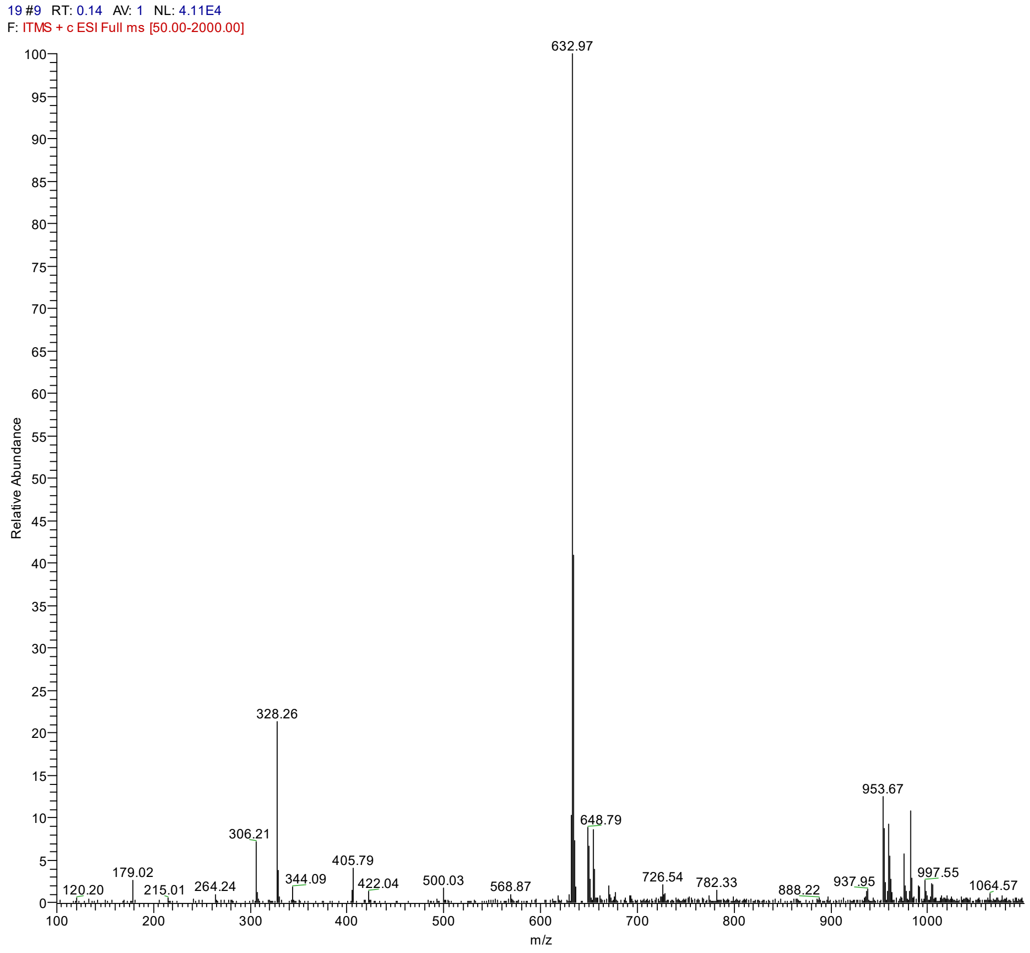

Figure S1 ESI-MS mass spectrum of **P.**

Figure S2

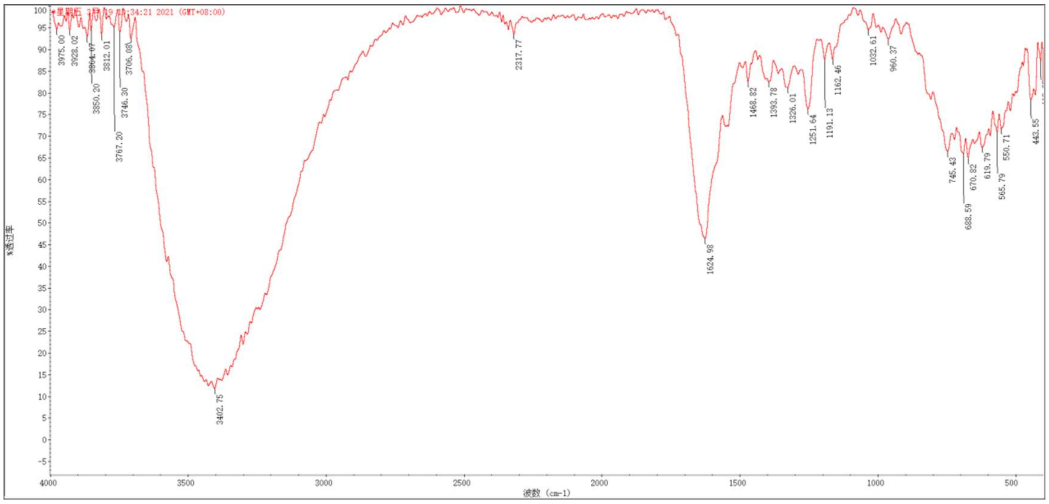

Figure S2 IR spectrum of **P.**

Figure S3

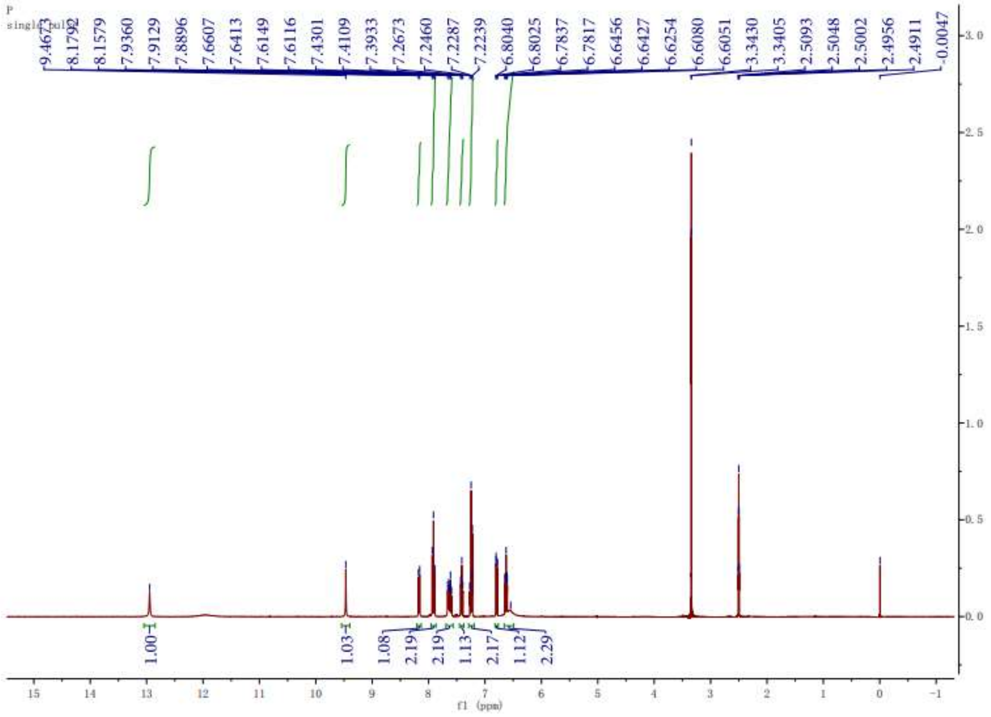

Figure S3 ^{1}H-NMR spectrum of **P.**

Figure S4

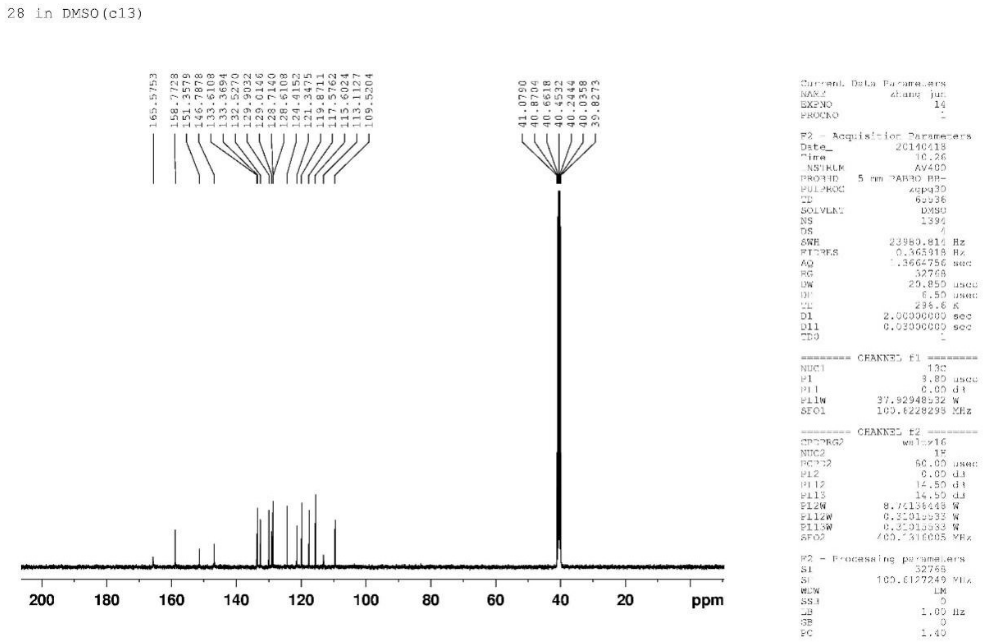

Figure S4 ^{13}C-NMR spectrum of **P.**

Figure S5

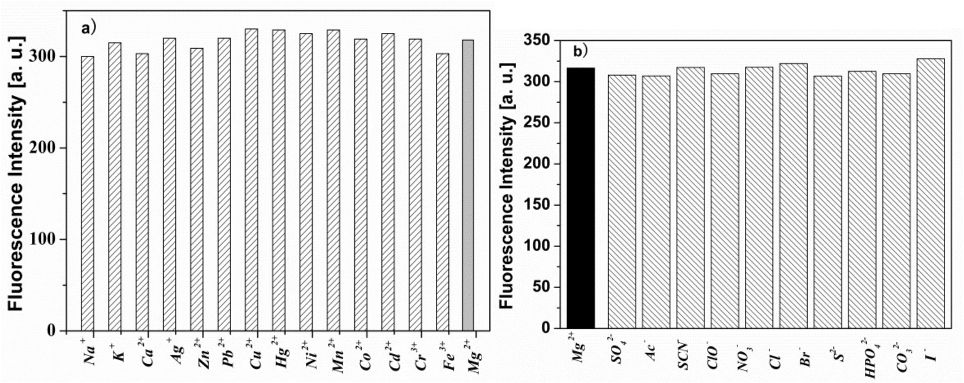

Figure S5 (**a**) fluorescence response of **P** (10 μM) to 10 μM of Mg^{2+} and to the mixture of 10 μM individual other metal ions with 10 μM of Mg^{2+}; (**b**) fluorescence response of **P** (10 μM) to 10 μM of Mg^{2+} and to the mixture of 10 μM individual anions with 10 μM of Mg^{2+}.

Figure S6

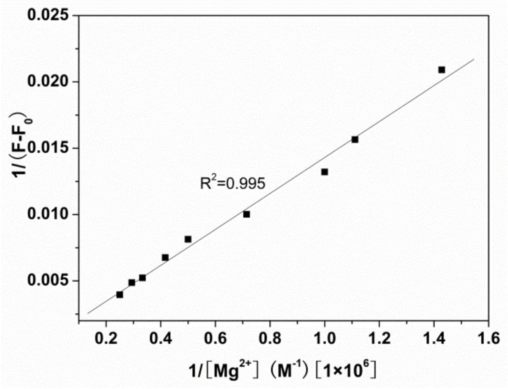

Figure S6 The plot of fluorescence intensity at 482 nm shows a linear relationship of $1/(F - F_0)$ vs $1/[Mg^{2+}]$, indicating the 1:1 stoichiometry of Mg^{2+} and probe **P.**

Figure S7

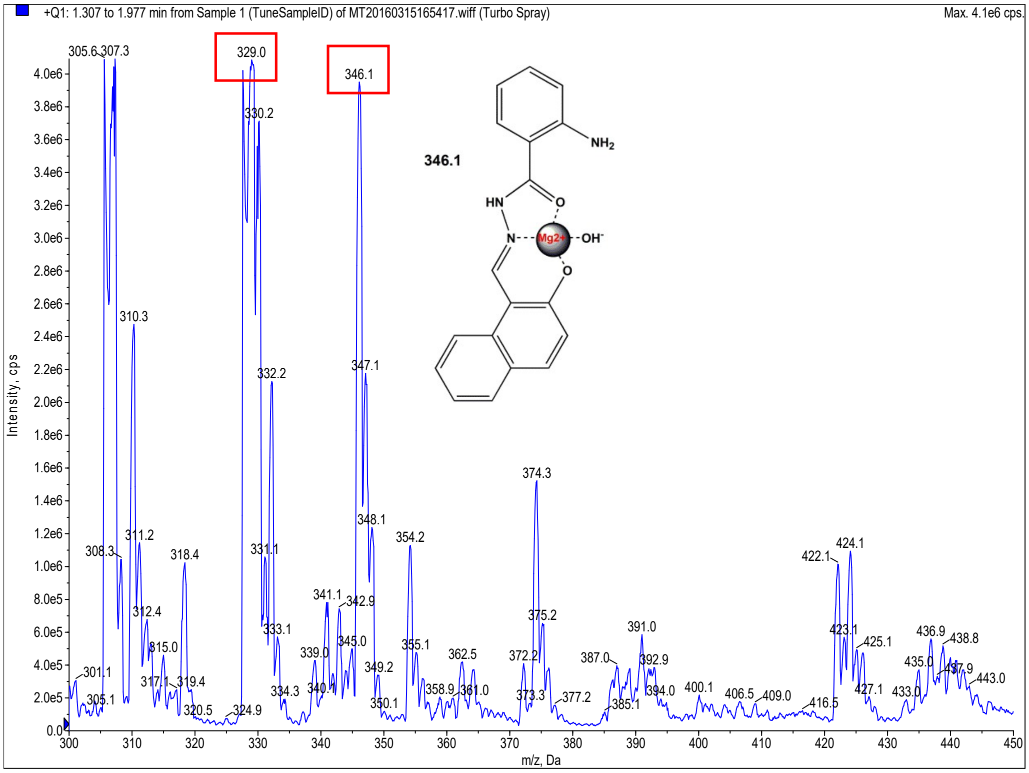

Figure S7 ESI-MS mass spectrum of **P** + Mg^{2+}.

Figure S8

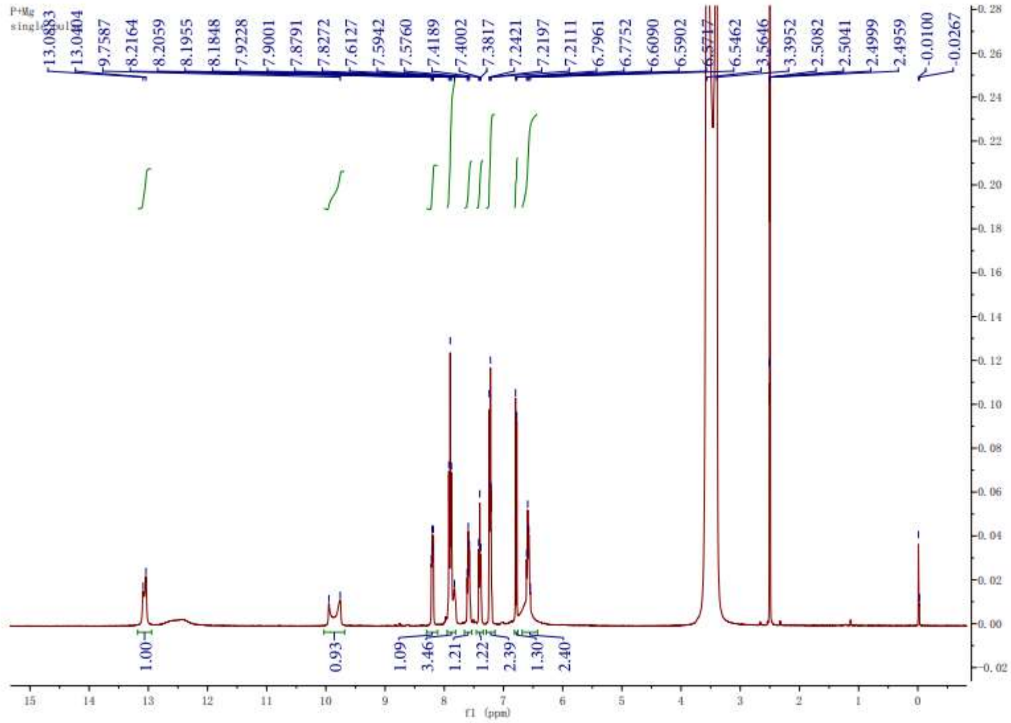

Figure S8 ^{1}H-NMR spectrum of **P + Mg^{2+}.**

Figure S9

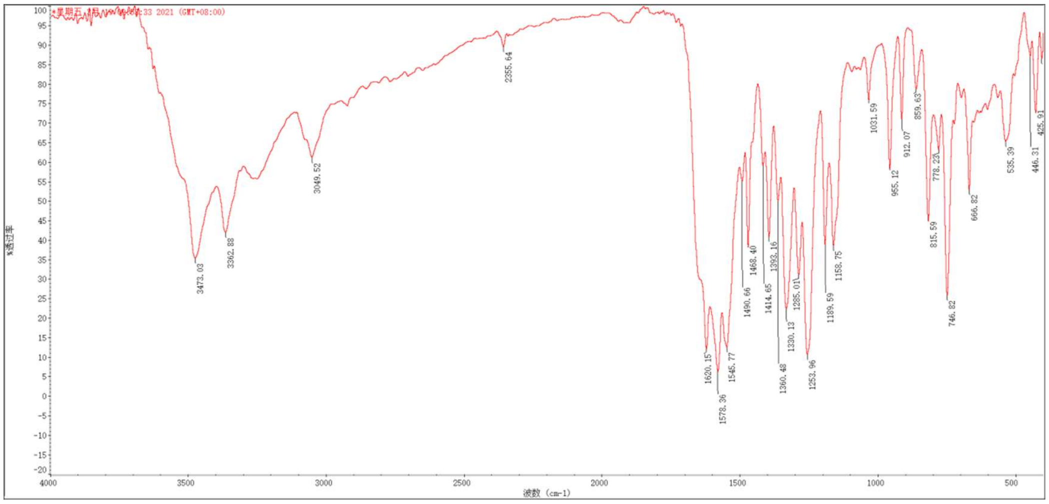

Figure S9 IR spectrum of **P + Mg^{2+}.**

Cytotoxicity assay

The in vitro cytotoxicity was measured by using the methyl thiazolyl tetrazolium (MTT) assay in Hl-7701 cells. Cells were seeded into 96-well cell culture plate at 4000 /well, cultured at 37 °C and 5 % CO_2 for 24 h, and then different concentrations of probe **P** (0, 0.1, 1, 10 μM) were added to the wells. The cells were then incubated for 48 h at 37 °C under 5% CO_2. Subsequently, 20 μL MTT (5 mg/mL) was added to each well and incubated for an additional 4 h at 37 °C under 5% CO_2. Cells were lysed in triple liquid (10 % SDS, 0.012 M HCl, 5 % isopropanol), and the amount of MTT formazan was qualified by determining the absorbance at 570 nm using a microplate reader (Tecan, Austria).

The following formula was used to calculate the inhibition of cell growth: Cell viability (%) = (mean of Abs. value of treatment group / mean Abs. value of control) • 100%.

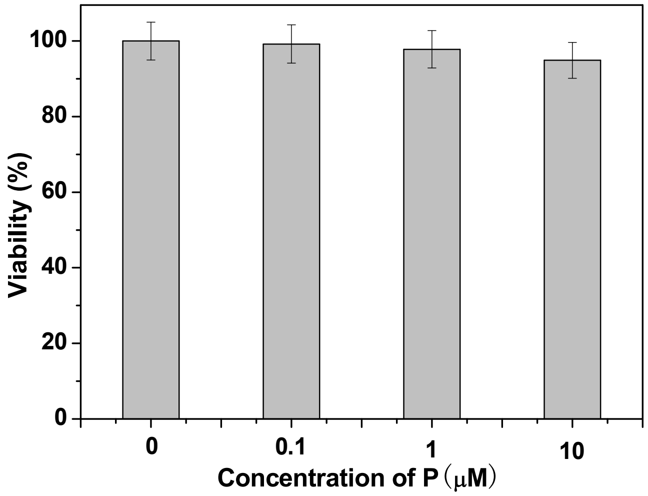

Figure S10 Cell viability values (%) estimated by MTT proliferation test versus incubation concentrations of **P**. Hl-7701 cells cells were cultured in the presence of 0−10 μM **P** at 37 °C.
